# Glycated Hemoglobin < 6.5% Is Associated With Uroseptic Shock in Diabetic Patients With Urinary Tract Infection

**DOI:** 10.3389/fmed.2020.515506

**Published:** 2020-12-01

**Authors:** Yi-Chien Lee, Tsung-Hsien Chen, Meng-Chang Hsiao, Peir-Haur Hung, Shao-Hsien Tung, Chih-Yen Hsiao

**Affiliations:** ^1^Department of Internal Medicine, Fu Jen Catholic University Hospital, Fu Jen Catholic University, New Taipei, Taiwan; ^2^School of Medicine, College of Medicine, Fu Jen Catholic University, New Taipei, Taiwan; ^3^Division of Nephrology, Department of Internal Medicine, Ditmanson Medical Foundation Chia-Yi Christian Hospital, Chiayi, Taiwan; ^4^Sema4, Brandford, CT, United States; ^5^Department of Applied Life Science and Health, Chia Nan University of Pharmacy and Science, Tainan, Taiwan; ^6^Department of Hospital and Health Care Administration, Chia Nan University of Pharmacy and Science, Tainan, Taiwan

**Keywords:** urinary tract infection, uroseptic shock, diabetes, glycated hemoglobin, congestive heart failure, bacteremia

## Abstract

This study aimed to compare the clinical characteristics and treatment outcomes of diabetic and non-diabetic individuals with urinary tract infection (UTI) and determine whether glycated hemoglobin (HbA1c) levels <6. 5% leads to uroseptic shock in diabetic individuals. We retrospectively collected and analyzed the clinical data of 1,363 individuals with UTIs in Taiwan from January 2006 to January 2018. Of the 345 diabetic individuals, 61 (17.7%) developed uroseptic shock. Diabetic patients who developed uroseptic shock tended to be older and males and, had a history of congestive heart failure, urolithiasis, higher serum creatinine level during hospitalization, lower serum HbA1c level, bacteremia, and acute kidney injury. Backward stepwise multivariate logistic regression analysis showed that male gender [odds ratio (OR), 1.861; 95% confidence interval (CI), 1.009–3.433; *P* = 0.047], congestive heart failure (OR, 4.036; 95% CI, 1.542–10.565; *P* = 0.004), bacteremia (OR, 2.875; 95% CI, 1.539–5.370; *P* = 0.001), and HbA1c level <6.5% (OR, 2.923; 95% CI, 1.580–5.406; *P* = 0.001) were associated with an increased risk of developing uroseptic shock among diabetic patients during hospitalization due to UTI. HbA1c level <6.5% is independently associated with uroseptic shock in diabetic patients with UTI.

## Introduction

Urinary tract infection (UTI) is one of the most prevalent infectious diseases in the general population, with an overall annual incidence of 17.5 per 1,000 population per year in Canada (1). Urosepsis is a severe complication of UTI, accounting for 20–30% of all septic patients (2). Furthermore, the globally accepted mortality rate of severe sepsis is 20–40% (3). A previous study identified several high risk factors for urosepsis including old age, female sex, diabetes, immunosuppressive status, use of chemotherapeutic agents or steroids, anemia, and chronic renal failure (4). Our previous study further identified that coronary artery disease (CAD), congestive heart failure (CHF), and acute kidney injury (AKI) are associated with uroseptic shock in patients with UTI (5).

Diabetes mellitus (DM) is one of the fastest growing public health problems in developing countries due to rapid urbanization and excessive caloric intake (6). Previous studies have shown that diabetic individuals are susceptible to UTI (7, 8), which can be attributed to a weak immune system (9, 10), poor metabolic control (11, 12), and inadequate bladder emptying due to autonomic neuropathy (13, 14). Glycated hemoglobin (HbA1c) measurement is the gold standard for the assessment of long-term glycemic control, and poor glycemic control contributes to the development of urosepsis (15). However, the impact of glycemic control on infection outcome, such as UTI with septic shock in diabetic individuals, is still unknown. Therefore, we aimed to compare the clinical characteristics and treatment outcomes of diabetic and non-diabetic individuals with UTI and to determine whether HbA1c level <6.5% is independently associated with uroseptic shock for diabetic individuals.

## Materials and Methods

### Study Design

This study included 1,363 patients diagnosed with community-onset UTI from Chia-Yi Christian Hospital in Taiwan from January 2006 to January 2018 and was approved by the ethics committee of the Ditmanson Medical Foundation Chia-Yi Christian Hospital (approval number: CYCH-IRB-2019061).

**Figure 1:**
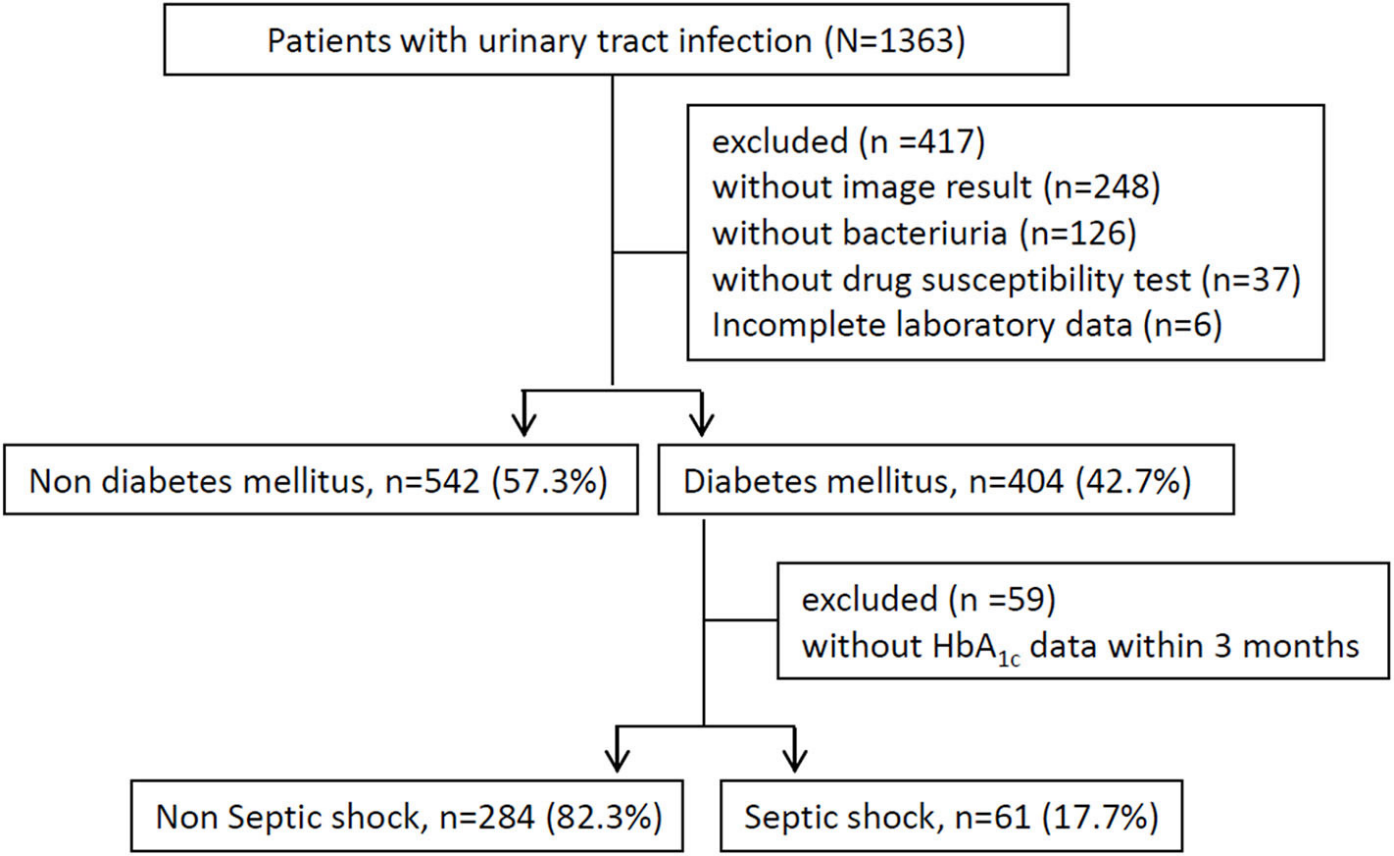
Flowchart of the inclusion and exclusion criteria of the study population.

The enrolled individuals had to meet the following criteria: (a) undergoing image survey, including ultrasound or computed tomography scans, (b) presence of a bacterial isolation of more than 10^5^ colony-forming units/mL from a urine specimen, (c) report of antimicrobial susceptibility tests, and (d) completion of required laboratory data (Figure 1). The subject assessments included the following: age, sex, mean white blood cell (WBC) count, platelet count, baseline estimated glomerular filtration rate (eGFR), HbA1c level, comorbidities [hypertension, CHF, CAD, stroke, chronic kidney disease (CKD), and liver cirrhosis history], indwelling Foley catheter, afebrile status during hospitalization, bacteremia, urolithiasis, hospitalized serum creatinine level, causative microorganisms (*Escherichia coli, Proteus* spp., *Klebsiella* spp., *Enterococcus* spp., *Pseudomonas* spp.), and antimicrobial resistance pattern. All patients would receive appropriate antibiotic treatment, i.e., broad-spectrum antimicrobial agents were prescribed for those with shock development at the time of admission or during hospitalization, and definite antibiotic therapy was administered based on available microbiological results. Among those patients complicated with abscess formation or hydronephrosis, invasive procedure or surgery will be performed if they agreed such management.

WBC and platelet counts and HbA1c level were measured using Sysmex XE-5000 hematology analyzer (Diamond Diagnostics, MA), and hospitalized serum creatinine level was measured using LABSPEC 008 (Hitachi).

### Definitions

Patients with an increased serum creatinine level equal to or more than twice the baseline were considered to have AKI (16, 17). Uroseptic shock was defined as sepsis with hypotension [systolic blood pressure (SBP) < 90 mmHg, mean arterial pressure < 70 mmHg, or a decrease in SBP by >40 mmHg in the absence of other causes of hypotension] over 1 h, despite adequate fluid resuscitation at the time of admission or during hospitalization (18). CKD was defined as eGFR < 60 ml/min/1.73 m^2^ for >3 months (19). Afebrile status was defined as a body temperature ≤38.3°C (101°F) during hospitalization. Multiple drug resistance (MDR) was defined as the resistance of isolates to at least three antimicrobial categories according to the international expert recommendations (20).

### Statistical Analyses

Data were expressed as mean ± SD and number (percentage). Baseline characteristics were compared between patients with and without DM or septic shock using Student's *t*-test for continuous variables and chi-square test for categorical variables. All potentially associated variables were tested using univariate analysis first. Factors associated with the development of uroseptic shock were identified using a backward stepwise multivariate logistic regression model. A linear regression model was used to identify the parameters that had collinearity, and these were not considered simultaneously in the final multivariate analysis. A *P*-value of <0.05 in a test of significance was considered statistically significant. All analyses were performed using the SPSS software for Windows (SPSS Inc, v. 17.0, Chicago, IL).

## Results

### Study Population

A total of 1,363 patients with UTI were recruited between January 2006 and January 2018. We excluded 248 patients without image results, 126 patients without bacterial growth from urine culture, 37 patients without an antibiotic susceptibility test, and 6 patients without complete laboratory data. The final study population comprised 946 patients (Figure 1). The mean age was 67 ± 17 years, 266 (28.1%) were male, and 404 (42.7%) had DM. Bacteremia, AKI, and uroseptic shock were identified in 434 (45.9%), 134 (14.2%), and 184 (19.5%) patients, respectively. MDR uropathogen infection was observed in 341 (36%) patients, and their baseline eGFR was 74.04 ± 29.94 mL/min/1.73 m^2^. Additionally, the most common pathogens isolated from the urine of diabetic patients with UTI were *Escherichia coli* (76.4%), *Klebsiella* spp. (7.6%), and *Pseudomonas* spp. (5.6%).

### Comparison of Clinical Characteristics Between Diabetic and Non-diabetic Individuals With Urinary Tract Infection (UTI)

The comparison of demographic, laboratory, and microbiological variables between 542 non-diabetic individuals and 404 diabetic individuals is shown in Table 1. Participants in the diabetic group had an older mean age (71 ± 12 vs. 64 ± 20 years, *P* < 0.001), lower baseline eGFR (67.47 ± 29.34 vs. 78.94 ± 29.46 mL/min/1.73 m^2^, *P* < 0.001), lower hemoglobin level (11.74 ± 2.20 vs. 12.05 ± 1.96 g/dl, *P* = 0.003), higher WBC count (13.93 ± 6.50 vs. 12.89 ± 5.89 10^3^/uL, *P* = 0.021), higher serum creatinine level at admission (1.89 ± 1.85 vs. 1.41 ± 1.30 mg/dL, *P* < 0.001), higher chance of developingbacteremia (51.5 vs. 41.7%, *P* = 0.003), and more isolates of *Klebsiella* spp. (10.1 vs. 5.7%, *P* = 0.011) than the participants in the non-diabetic group. Diabetic participants were also more likely to have a history of hypertension (67.8 vs. 40%, *P* < 0.001), CAD (15.6 vs. 7.4%, *P* < 0.001), stroke (27.5 vs. 17.7%, *P* < 0.001), CKD (42.1 vs. 28.6%, *P* < 0.001), and liver cirrhosis (8.7 vs. 4.8%, *P* = 0.017) compared with non-diabetic participants. The all cause in-hospital mortality was slightly higher in diabetic patients than in non-diabetic patients without significant difference (0.7 vs. 0.6%, *P* = 0.705) (Table 1).

**Table 1:** Characteristics of hospitalized patients with urinary tract infection with and without diabetes mellitus.

**Characteristic**	**All (*n* = 946)**	**Diabetes mellitus (DM)**		***P*-value**
		**Non-DM (*n* = 542)**	**DM (*n* = 404)**	
Age (year)	67 ± 17	64 ± 20	71 ± 12	<0.001^*^
Sex (male)	266 (28.1)	148 (27.3)	118 (29.2)	0.520^¥^
Hypertension	491 (51.9)	217 (40.0)	274 (7.8)	<0.001^¥^
Congestive heart failure	47 (5.0)	26 (4.8)	21 (5.2)	0.779^¥^
Coronary artery disease	103 (10.9)	40 (7.4)	63 (15.6)	<0.001^¥^
Stroke	207 (21.9)	96 (17.7)	111 (27.5)	<0.001^¥^
Chronic kidney disease	325 (34.4)	155 (28.6)	170 (42.1)	<0.001^¥^
Liver cirrhosis	61 (6.4)	26 (4.8)	35 (8.7)	0.017^¥^
Urolithiasis	168 (17.8)	93 (17.2)	75 (18.6)	0.576^¥^
Indwelling Foley catheter	69 (7.3)	41 (7.6)	28 (6.9)	0.711^¥^
Afebrile	366 (38.7)	199 (36.7)	167 (41.3)	0.149^¥^
Bacteremia	434 (45.9)	226 (41.7)	208 (51.5)	0.003^¥^
Septic shock	184 (19.5)	113 (20.8)	71 (17.6)	0.208^¥^
Acute kidney injury	134 (14.2)	68 (12.5)	66 (16.3)	0.098^¥^
All-cause mortality	6 (0.6)	3 (0.6)	3 (0.7)	0.705^¥^
Baseline eGFR (mL/min/1.73 m^2^)	74.04 ± 29.94	78.94 ± 29.46	67.47 ± 29.34	<0.001^*^
Hospitalized serum creatinine (mg/dL)	1.62 ± 1.57	1.41 ± 1.30	1.89 ± 1.85	<0.001^*^
White blood cell (10^3^/uL)	13.33 ± 6.18	12.89 ± 5.89	13.93 ± 6.50	0.021^*^
Hemoglobin (g/dl)	11.92 ± 2.07	12.05 ± 1.96	11.74 ± 2.20	0.003^*^
Platelet count (10^3^/uL)	203.78 ± 115.66	198.57 ± 80.75	210.78 ± 150.10	0.944^*^
Multiple drug resistance pathogen	341 (36.0)	193 (35.6)	148 (36.6)	0.745^¥^
*Escherichia coli*	723 (76.4)	426 (78.6)	297 (73.5)	0.068^¥^
*Proteus* spp.	33 (3.5)	17 (3.1)	16 (4.0)	0.495^¥^
*Klebsiella* spp.	72 (7.6)	31 (5.7)	41 (10.1)	0.011^¥^
*Pseudomonas* spp.	53 (5.6)	33 (6.1)	20 (5.0)	0.452^¥^
*Enterococcus* spp.	36 (3.8)	19 (3.5)	17 (4.2)	0.576^¥^

* *Student's t-test or Mann-Whitney U-test*.

¥ *chi-square test*.

*Data are expressed as mean ± standard deviation or number (percentage)*.

*eGFR, estimated glomerular filtration rate*.

### Factors Related to Uroseptic Shock in Diabetic Patients With UTI

Regarding the 404 diabetic patients with UTI, 59 were excluded from the analysis of uroseptic shock because they had insufficient HbA1c data 3 months prior to admission. The overall uroseptic shock rate was 17.7% (61/345), and the demographic and clinical characteristics of diabetic patients presenting with or without uroseptic shock are shown in Table 2. Diabetic patients with uroseptic shock were older (74 ± 11 vs. 70 ± 13 years, *P* = 0.013), had higher serum creatinine level during hospitalization (2.48 ± 2.23 vs. 1.89 ± 1.87 mg/dL, *P* = 0.033), had lower serum HbA1c level (7.4 ± 2.1% vs. 8.1 ± 2.0%, *P* = 0.016), were more predominantly men (41 vs. 28.2%, *P* = 0.048), had higher chance of developing bacteremia (67.2 vs. 47.5%, *P* = 0.005) and urolithiasis (29.5 vs. 15.8%, *P* = 0.012), and were more likely to have a past history of CHF (14.8 vs. 4.2%, *P* = 0.005) and to experience AKI (37.7 vs. 13.7%, *P* < 0.001) compared with diabetic patients without uroseptic shock. A higher prevalence of low HbA1c level (<6.5%) was observed in the uroseptic shock group compared to the non-uroseptic shock group (42.6 vs. 20.4%, *P* < 0.001). The all-cause in-hospital mortality was slightly higher in uroseptic shock patients than in non-uroseptic shock patients without significant difference (3.3 vs. 0.4%, *P* = 0.082). Backward stepwise multivariate logistic regression analysis demonstrated that male gender [odds ratio (OR), 1.861; 95% confidence interval (CI), 1.009–3.433; *P* = 0.047], CHF (OR, 4.036; 95% CI, 1.542–10.565; *P* = 0.004), bacteremia (OR, 2.875; 95% CI, 1.539–5.370; *P* = 0.001), and HbA1c level <6.5% (OR, 2.923; 95% CI, 1.580–5.406; *P* = 0.001) were the significant predictors for the development of uroseptic shock in diabetic patients with UTI (Table 3).

**Table 2:** Characteristics of hospitalized patients with urinary tract infection with and without septic shock.

**Characteristic**	**All (*n* = 345)**	**Septic shock**		***P*-value**
		**No (*n* = 284)**	**Yes (*n* = 61)**	
Age (year)	71 ± 13	70 ± 13	74 ± 11	0.013x^*^
Sex (male)	105 (30.4%)	80 (28.2)	25 (41.0)	0.048^¥^
Hypertension	229 (66.4)	188 (66.2)	41 (67.2)	0.879^¥^
Congestive heart failure	21 (6.1)	12 (4.2)	9 (14.8)	0.005^¥^
Coronary artery disease	56 (16.2)	42 (14.8)	14 (23.0)	0.117^¥^
Stroke	100 (29.0)	84 (29.6)	16 (26.2)	0.601^¥^
Chronic kidney disease	152 (44.1)	124 (43.7)	28 (45.9)	0.749^*^
Liver cirrhosis	30 (8.7)	23 (8.1)	7 (11.5)	0.396^*^
Urolithiasis	63 (18.3)	45 (15.8)	18 (29.5)	0.012^¥^
Indwelling Foley catheter	27 (7.8)	23 (8.1)	4 (6.6)	0.799^¥^
Afebrile	147 (42.6)	123 (43.3)	24 (39.3)	0.570^¥^
Bacteremia	176 (51.0)	135 (47.5)	41 (67.2)	0.005^¥^
Acute kidney injury	62 (18.0)	39 (13.7)	23 (37.7)	<0.001^*^
All-cause mortality	3 (0.9)	1 (0.4)	2 (3.3)	0.082^¥^
Baseline eGFR (mL/min/1.73 m^2^)	66.17 ± 29.93	66.63 ± 30.73	64.00 ± 25.99	0.533^*^
Hospitalized serum creatinine (mg/dL)	1.99 ± 1.95	1.89 ± 1.87	2.48 ± 2.23	0.033^*^
White blood cell (10^3^/uL)	14.01 ± 6.46	13.81 ± 6.25	14.89 ± 7.38	0.238^*^
Hemoglobin (g/dl)	11.67 ± 2.23	11.67 ± 2.23	11.70 ± 2.29	0.917^*^
Platelet count (10^3^/uL)	210.07 ± 154.99	213.87 ± 163.9	192.38 ± 103.08	0.327^*^
HbA1c (%)	8.0 ± 2.0	8.1 ± 2.0	7.4 ± 2.1	0.016^*^
HbA1c (%)				<0.001^¥^
<6.5 (%)	84 (24.3)	58 (20.4)	26 (42.6)	
≥6.5 (%)	261 (75.7%)	226 (79.6)	35 (57.4)	
Multiple drug resistance pathogen	128 (37.1)	99 (34.9)	29 (47.5)	0.063^¥^
*Escherichia coli*	253 (73.3)	205 (72.2)	48 (78.7)	0.297^¥^
*Proteus* spp.	12 (3.5)	10 (3.5)	2 (3.3)	1.000^¥^
*Klebsiella* spp.	37 (10.7)	33 (11.6)	4 (6.6)	0.246^¥^
*Enterococcus* spp.	14 (4.1)	11 (3.9)	3 (4.9)	0.720^¥^
*Pseudomonas* spp.	15 (4.3)	14 (4.9)	1 (1.6)	0.486^¥^

* *Student's t-test*.

¥ *chi-square test or Fisher's exact test*.

D*ata are expressed as mean ± standard deviation or number (percentage)*.

*eGFR, estimated glomerular filtration rate; HbA1c, Glycated hemoglobin*.

**Table 3:** Backward stepwise multivariate logistic regression analyses of factors related to uroseptic shock in diabetic patients with urinary tract infection.

**Covariate**	**Multivariate**	
	**OR (95% CI)**	***P*-value**
Sex (male)	1.861 (1.009–3.433)	0.047
Congestive heart failure	4.036 (1.542–10.565)	0.004
Bacteremia	2.875 (1.539–5.370)	0.001
HbA1c <6.5 (%)	2.923 (1.580–5.406)	0.001

*HbA1c, Glycated hemoglobin*.

*Backward stepwise model includes age, sex, hypertension, congestive heart failure, coronary artery disease, stroke, chronic kidney disease, liver cirrhosis, urolithiasis, indwelling Foley catheter, afebrile, bacteremia, white blood cell, hemoglobin, platelet counts, HbA1c < 6.5 (%), multiple drug resistance pathogen*.

## Discussion

This study was conducted to assess the clinical characteristics of diabetic and non-diabetic individuals with UTI and to investigate the risk factors for uroseptic shock in diabetic patients. We found that patients in the diabetic group have distinct clinical manifestations of older age and multiple comorbidities. They were also associated with worse laboratory profiles, including the development of bacteremia, increased isolates of *Klebsiella* spp., impaired renal function tests, lower hemoglobin level, and higher WBC count. Male gender, CHF, bacteremia, and HbA1c level < 6.5% were independently associated with uroseptic shock in diabetic patients.

Consistent with Kim et al.'s findings (21), in this study, higher median age with more comorbidities and worsening laboratory parameters indicating severe disease were frequently observed in the diabetic group. Additionally, Al-Rubeaan et al. (22) found that hypertension is associated with the occurrence of UTI in diabetic patients. Accordingly, we should cautiously monitor the inflammatory markers, including WBC count, if elderly diabetic patients with multiple underlying diseases present to clinicians with UTI. To the best of our knowledge, individuals with diabetes knownare significantly associated with more serious manifestations of UTI (23, 24), and the development of UTI in diabetic patients can lead to severe kidney damage and renal failure (25), which were also demonstrated in our results. Therefore, physicians should pay careful attention to the potential deterioration of UTI in diabetic patients.

Several important risk factors including male gender, CHF, bacteremia, and HbA1c level <6.5% for uroseptic shock among diabetic patients with UTI have been identified in this study. Consistent with our previous study (5), patients with an underlying disease of CHF would be more likely to experience a decline in cardiac contractility and myocardial dysfunction and to experience mitochondrial impairment and apoptosis in sepsis-induced cardiomyopathy (26), predisposing these patients to the development of uroseptic shock. Furthermore, the following three principal cardiovascular events are frequently observed during the progression of sepsis to severe sepsis or even septic shock, particularly in individuals with CHF: depletion of intravascular volume due to capillary leak, depressed vascular tone, and impaired cardiac contractility (27). Accordingly, aggressive fluid resuscitation with the administration of broad-spectrum antimicrobial agents should be recommended for diabetic patients with CHF presenting with UTI.

Serious infection and higher incidence of bacteremic infections had been reported among those male patients (28, 29), and increase in anti-inflammatory mediators in females and female sex steroid strengthening humoral immune responses might contribute to the better outcome (30), which echoed our findings. Bacteremia is the presence of viable bacteria in the blood, which might trigger the development of sepsis with subsequent progression to septic shock (31). Previous studies have demonstrated that 26–33% of patients with bacteremic UTI present with uroseptic shock (32, 33) (35.2% in Taiwan) (34). Moreover, consistent with the present study, Ko et al. indicated that higher bacteremia rates were observed in patients with uroseptic shock induced by urolithiasis-related acute pyelonephritis (35), and bacteremia was identified as an independent risk factor for septic shock in patients with acute obstructive pyelonephritis (36). Consequently, prolonged duration of antibiotic therapy should be considered in diabetic patients with bacteremic UTI and in patientswith septic shock due to the existence of bacteremia accompanied with more serious manifestations.

Some physiological and pathogenic conditions, including hemolytic anemia, cirrhosis, CKD, and certain hemoglobinopathies, may result in lower HbA1c values due to the reduction in the number of glycosylated red blood cells (37). In the present study, none of the patients with hemoglobin variants were enrolled, and the distribution of anemia, liver cirrhosis, and CKD between the two groups with or without uroseptic shock had no statistical difference, indicating that the measured HbA1c values reflected the actual glycemia status within every participant without any confounding factors. The question of whether HbA1c affects the development of serious infection or mortality has been long debated. Contrary to our findings, some studies reported that a higher plasma level of HbA1c, independently predicting hospital mortality in diabetic patients with sepsis (38), was observed in patients with bloodstream infection (39) and that the use of intensive insulin treatment might be the cause. Currently, insulin therapy is a critical part of treatment for diabetic patients, particularly for those with severe sepsis or septic shock. Insulin promotes glucose uptake by cells, and appropriate concentrations of insulin are necessary for neutrophil function (40, 41).

Furthermore, the anti-inflammatory and other immunologic effects, including suppression of excess inflammation and improvement of macrophage function by insulin, have a clinical benefit in improving sepsis outcomes (42, 43). However, strict glycemic control increases the risk of hypoglycemia associated with subsequent mortality among critically ill patients with severe sepsis and septic shock (44). In our study, diabetic patients with HbA1c levels < 6.5% are less commonly taking insulin therapy than those with HbA1c ≥ 6.5% (9.5 vs. 30.3%, *P* < 0.001) to prevent hypoglycemia. Therefore, diabetic patients with HbA1c levels < 6.5% are also less likely to obtain the clinical benefit of insulin than diabetic patients with HbA1c levels ≥ 6.5%. The current study has identified that low HbA1c level is an independent risk factor associated with uroseptic shock in diabetic patients, but further investigation regarding the exact pathogenesis of HbA1c is required.

This study has several limitations. First, this is a retrospective study, the information about the patient's drug history, such as oral antidiabetic drugs and insulin, was incomplete. Further study with comprehensive drug history is warranted to find the impact of oral antidiabetic drugs, such as sodium glucose cotransporter 2 inhibitors, or insulin on the development of uroseptic shock in diabetic patients. Second, this single-center study which limits the generalizability of the results; hence, our findings might be not suitable for some other places. Third, we did not analyze the change in sepsis-related cytokines at different plasma levels of HbA1c or at variable concentrations of blood glucose, which could be the actual mechanism for the development of uroseptic shock induced by glycemic control. Fourth, this is a 12-year study, and most of the clinical data information were obtained before 2016. The parameters based on Sepsis-3 definition were not collected completely; hence, further studies are required to confirm our findings. Finally, considering that the mortality rate for patients with UTI is low, it may not be possible to accurately identify the risk factors associated with mortality by multivariate analysis. Regardless of these limitations, clinicians should pay careful attention on HbA1c level as it may be related to the development of uroseptic shock in diabetic patients. Hence, intensive insulin treatment should be judiciously provided to patients with uroseptic shock regardless of the HbA1c level.

In summary, different clinical manifestations were evident between patients with UTI with and without diabetes, and the former was more common in old age with more comorbidities and worse laboratory profiles. HbA1c level <6.5% was independently associated with uroseptic shock in diabetic patients. When we treat critically ill patients, specifically uroseptic patients with low HbA1c level, the prudent use of insulin should be considered to avoid poor outcomes.

## Data Availability Statement

The datasets generated for this study are available on request to the corresponding author.

## Ethics Statement

This study was conducted in concordance with institutional patient safety laws and has been approved by the Institutional Review Board of Chiayi Christian Hospital (approval no. CYCH-IRB-2019061). This study was performed in accordance with the Declaration of Helsinki. The patients/participants provided their written informed consent to participate in this study.

## Author Contributions

All authors participated in the interpretation of the studies and analysis of the data and also reviewed and approved the final version of the manuscript. Y-CL and C-YH: protocol/project development. Y-CL, C-YH, and S-HT: data collection or management. Y-CL and T-HC: manuscript writing/editing. T-HC and C-YH: data analysis. M-CH, P-HH, and C-YH: manuscript review. C-YH: scientific advisor.

## Conflict of Interest

M-CH was employed by Sema4. The remaining authors declare that the research was conducted in the absence of any commercial or financial relationships that could be construed as a potential conflict of interest.
